# Spinal Coccidioidomycosis: A Complication From Medication Noncompliance

**DOI:** 10.7759/cureus.9304

**Published:** 2020-07-20

**Authors:** Surav M Sakya, Judy P Sakya, David R Hallan, Irfan Warraich

**Affiliations:** 1 Medicine, Penn State College of Medicine, Hershey, USA; 2 Medicine, Texas Tech University Health Sciences Center, Lubbock, USA; 3 Neurosurgery, Penn State Health Milton S. Hershey Medical Center, Hershey, USA; 4 Pathology, Texas Tech University Health Sciences Center, Lubbock, USA

**Keywords:** spinal coccidioidomycosis, arachnoid adhesion, fluconazole, noncompliance

## Abstract

Spinal coccidioidomycosis is a rare disseminated form of coccidioidomycosis infection. According to the literature, majority of patients are African American males. We present a rare case of spinal coccidioidomycosis in a young, Caucasian female with coccidioidomycosis meningitis at age 16 years who presented with bilateral lower extremity weakness after antifungal medication lapse for one year. Imaging revealed cystic arachnoid formations along her thoracic spine. Pathology report confirmed spinal arachnoiditis with coccidioidomycosis. This case report details a rare incidence of spinal coccidioidomycosis and reviews previous literature.

## Introduction

Spinal coccidioidomycosis is a rare condition caused by hematogenous systemic dissemination of Coccidioides species. Coccidioidomycosis, a fungal infection commonly known as Valley fever, is endemic to the southwestern region of the United States. This respiratory illness, generally caused by fungal organisms Coccidioides immitis or Coccidioides posadasii, often presents as community-acquired pneumonia with fever and cough [1]. In immunocompetent individuals, this fungal disease is self-limiting; however, pulmonary and extrapulmonary symptoms, including meningitis, may present in immunocompromised individuals. Furthermore, individuals may develop dissemination of the fungal infection involving the musculoskeletal system, preferentially the axial bones, such as the vertebral bodies [2]. Spinal coccidioidomycosis involving one or more segments of spine may result in discitis, paravertebral soft tissue infection, vertebral body erosion and neural compression. Prompt diagnosis and treatment are necessary.

In the literature, several cases involving spinal coccidioidomycosis have been reported. However, these cases mainly involve African American men. Here, we present a rare case of a spinal coccidioidomycosis in a 24-year-old, Caucasian female. More specifically, our patient had arachnoiditis with coccidioidomycosis along the thoracic spine after one year of antifungal noncompliance. To the best of our knowledge, this is a rare case of spinal coccidioidomycosis in a young, Caucasian female, and highlights the importance of educating those on chronic antifungal therapy to never have lapses in medication compliance.

## Case presentation

**Figure 1:**
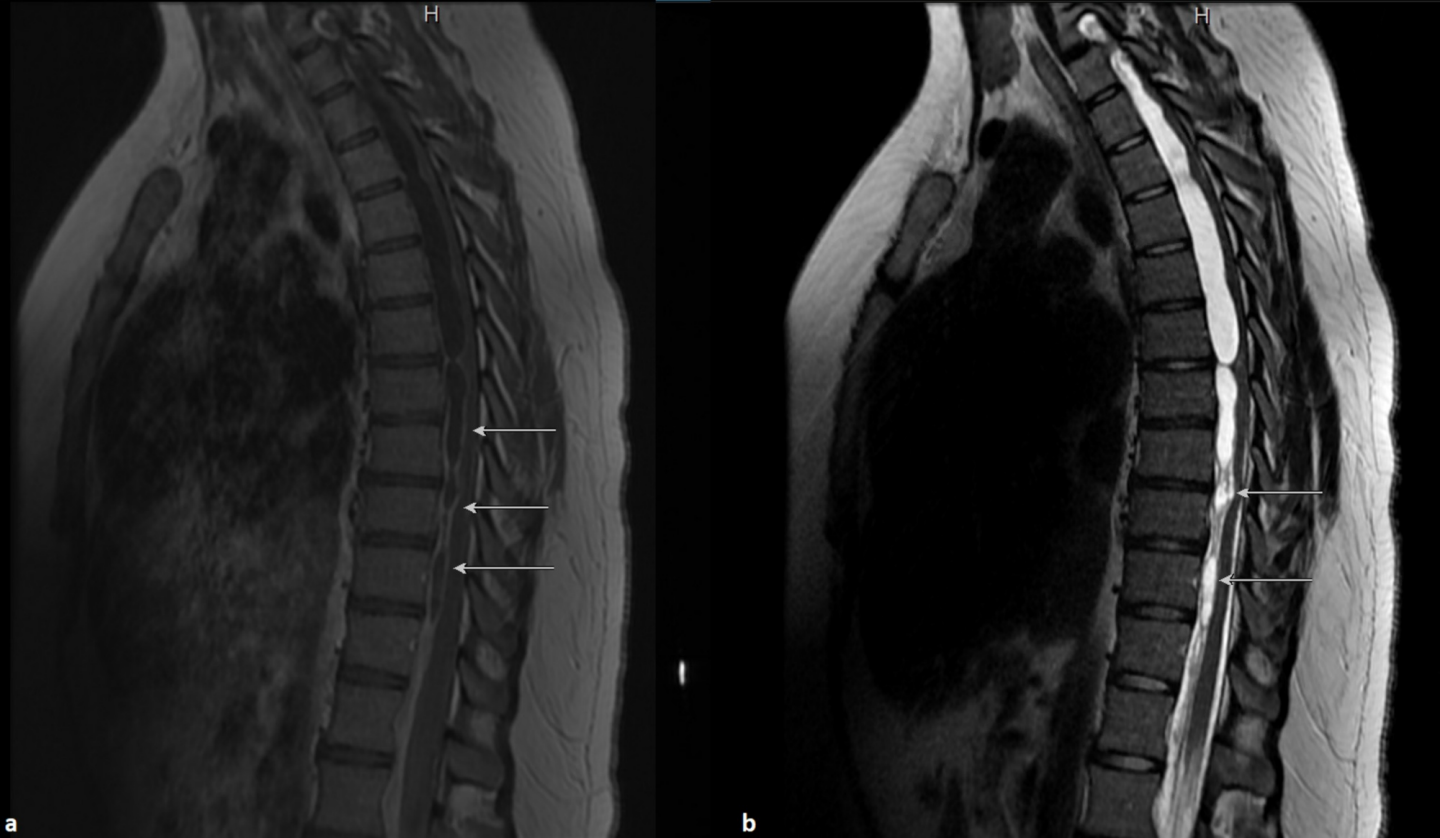
(a) T1 and (b) T2 sagittal thoracic MRI demonstrating cystic arachnoid formations (white arrows) from upper to lower thoracic spine.

An HIV-negative 24-year-old Caucasian female born and raised in New Mexico presented with worsening bilateral lower extremity weakness and numbness. The patient had been previously diagnosed with coccidioidomycosis meningitis at age 16 years and was on daily antifungal medication. She denied any fever, chills, dysuria and upper extremity weakness. She had stopped taking her regular antifungal medication for the past year to "see what would happen." The decision to stop the antifungal was not due to adverse effects, but her hopeful curiosity for meningitis remission without continued treatment. Physical exam revealed 3/5 motor strength in both proximal and distal lower extremity bilaterally and cold and vibration sensory deficits starting distally and increasing proximally. Cerebrospinal fluid (CSF) was negative for malignancy. Moreover, CSF and serum complement fixed Ab were positive for coccidioidomycosis (1:128 titer and 1:16 titer, respectively), and IgM and IgG were both positive. Her thoracic MRI showed enhancing septations within the thecal sac in the thoracic region, resembling cystic arachnoid formations (Figure 1).

**Figure 2:**
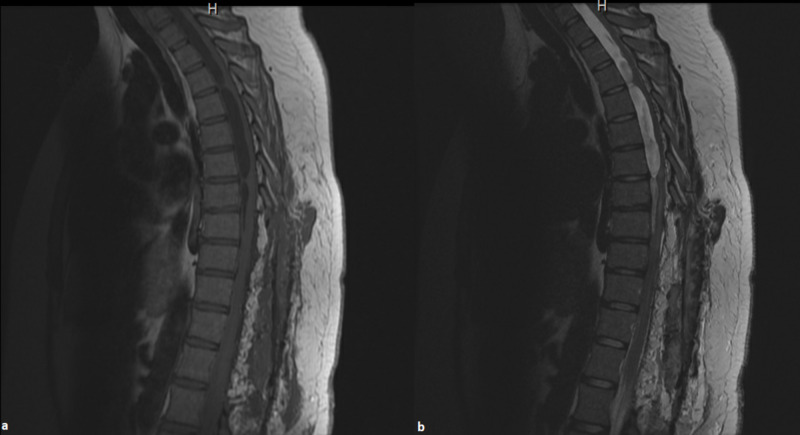
(a) T1 and (b) T2 sagittal thoracic MRI demonstrating postsurgical changes of laminectomy from T8 to L2. Arachnoid formations in the lower thoracic spine from T8 to T12 are no longer visualized.

Neurosurgery performed T8-L2 laminectomy for removal of arachnoid cysts (Figure 2).

**Figure 3:**
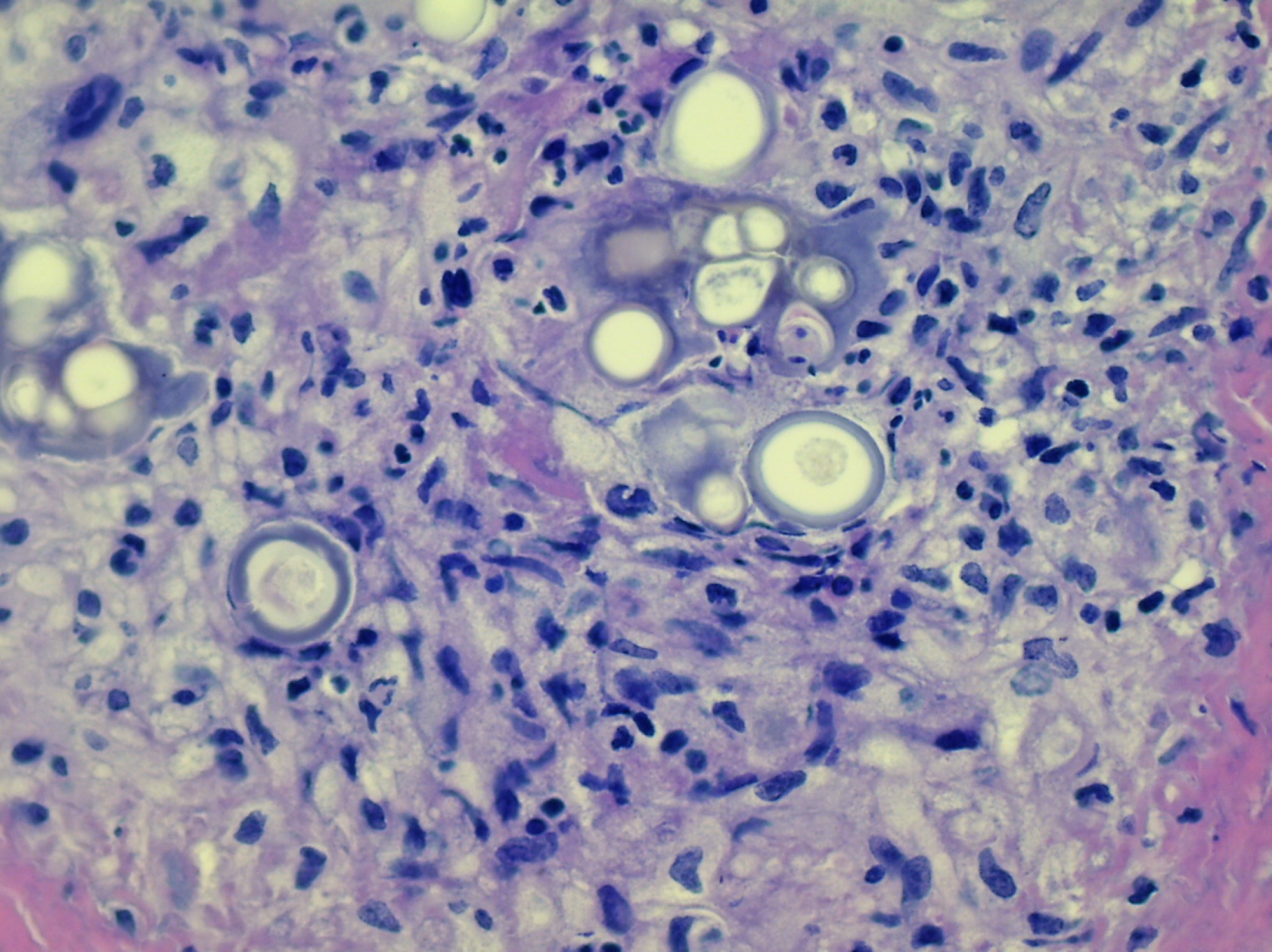
Histological features of arachnoiditis with coccidioidomycosis in a 24-year-old female patient. Cocci spherules surrounded by granulomatous inflammation are shown (H&E ×200).

**Figure 4:**
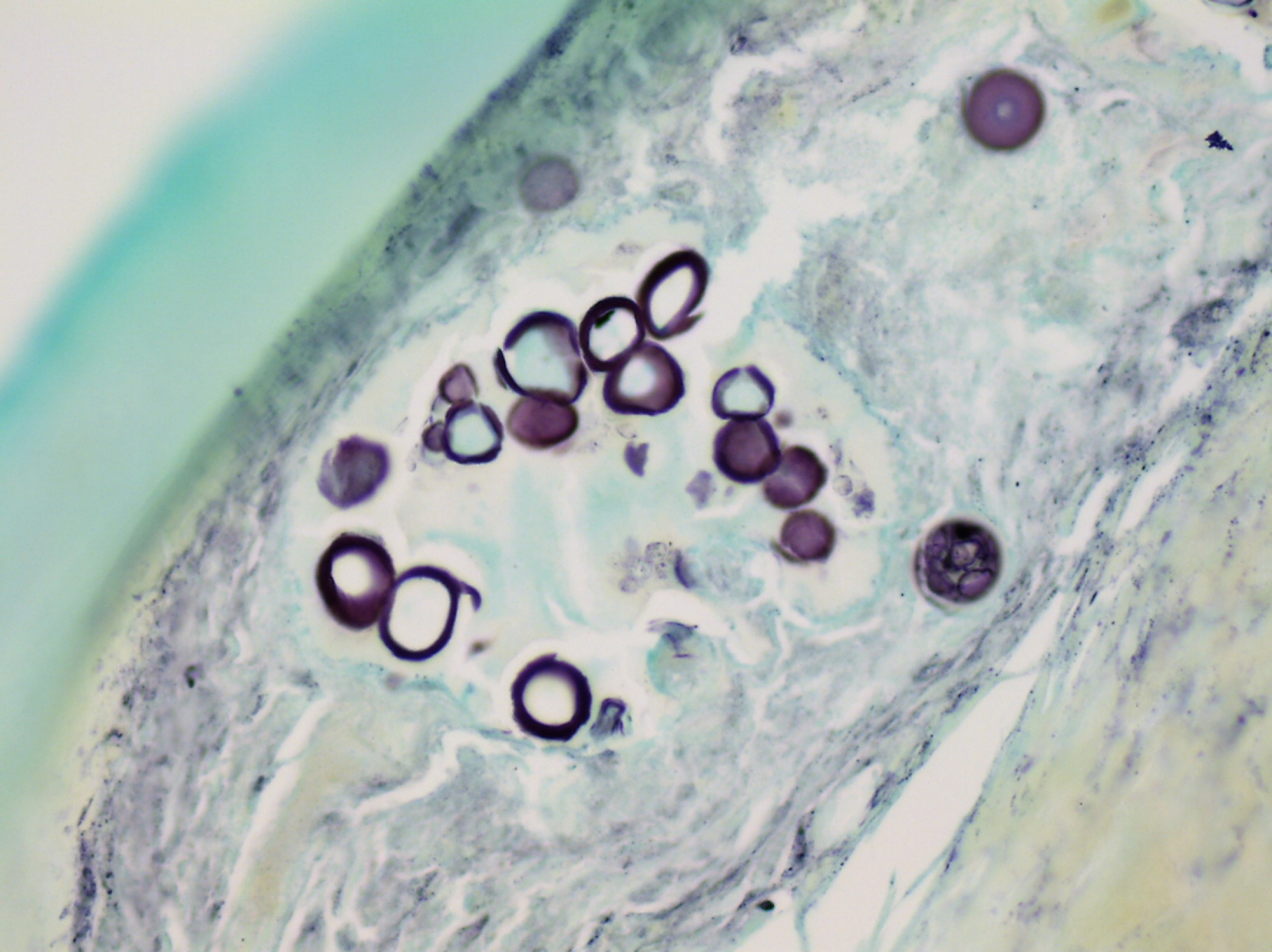
Histological features of arachnoiditis with coccidioidomycosis in a 24-year-old female patient. Cocci spherules are shown (Grocott methenamine silver ×200).

The pathology report from the biopsy of the arachnoiditis found chronic inflammation with coccidioidomycosis (Figures 3, 4).

The patient later was discharged on fluconazole 800 mg PO daily and started rehab. On a return visit a month later, she endorsed mild improvement of all her neurological deficits. The patient is encouraged to continue rehab and medication adherence.

## Discussion

Spinal coccidioidomycosis from infiltration of Coccidioides species in the vertebra can present with neurological manifestations [2,3]. Literature describes individuals with this disseminated infection presenting most commonly with back pain, neck pain, radiculopathy, sensory disturbances and paraparesis [1]. With these nonspecific symptoms, diagnosing spinal coccidioidomycosis can be challenging, but there are clues. For our patient, there was suspicion for coccidioidomycosis infection because of her previous history of coccidioidomycosis meningitis and labs showing positive antibody titers for coccidioidomycosis. In the literature, IgM elevation occurs within 1-3 weeks after symptom onset, and IgG elevation occurs 2-28 weeks after symptom onset. Antibodies over 1:16 indicate a disseminated disease [1,3]. Therefore, the patient’s medical history for previous coccidioidomycosis infection and patient’s residence are important considerations.

Furthermore, in a paper by Martirosyan et al., 164 cases of spinal coccidioidomycosis were obtained through a literature search from the National Library of Medicine between 1964 and 2014, yielding 24 papers. The average age range affected was 21 to 30 years, with most cases between 11 and 50 years. Out of 164 cases, 131 were males and 79 of the patients were African Americans. Most patients were not immunocompromised, and only 57 of the 164 cases were cases from coccidioidomycosis endemic areas [1]. From a paper by Ramanathan et al., three cases of spinal coccidioidomycosis from a retrospective review of medical records at a single institution from 2009 to 2018 were men [2]. Additionally, a paper by Martinez-Del-Campo et al. looked at all cases between 1977 and 2014 which totaled 140 cases. Patients had an average age of 36.2 years, and there were 95% males and 52% African Americans [3]. In the literature, only primary spinal coccidioidomycosis cases are reported; these cases are not from those with past medical history of coccidioidomycosis infection and from antifungal medication noncompliance. Although it is more likely to present in African American males, our young, Caucasian female patient was an exception. Most cases in the literature are of patients who traveled to an endemic area, but our patient had been living in an endemic area for years and had been exposed to coccidioidomycosis without being in an immunocompromised state.

Medical management guidelines institute lifelong therapy of azole therapy as treatment after an episode of meningitis. Without treatment, death occurs in 95% of individuals within two years. It is important to recognize and treat musculoskeletal coccidioidomycosis immediately as the mortality rate ranges from 10% to 16% [3]. For patients with a history of coccidioidomycosis meningitis, lifelong antifungal therapy is recommended, specifically azoles [4]. A paper by Dewsnup et al. conducted a study of 18 patients who had stopped azole therapy due to presumed cure. Out of 18 people, 14 had relapse from 0.5 to 30 months [5]. In our case, we had a patient with a rare spinal dissemination as a complication of medication noncompliance. Since the patient has been compliant for many years prior, it is surprising to present with spinal coccidioidomycosis. Moreover, there is lack of literature showing detailed complications from stopping long-term antifungal therapy. Our case stresses the importance of continued antifungal treatment without any lapses and details a complication from medication noncompliance.

## Conclusions

Even though most patients are African American males, it is important to recognize the importance of medical history and place of residence for prompt diagnosis and treatment of spinal coccidioidomycosis. Moreover, her condition was complicated by her medication noncompliance, and proper education is needed especially in young adults who must take daily medications. The importance of the patient remaining on lifelong antifungal therapy after coccidioidomycosis meningitis should be stressed, even after several years without recurrence, and dissemination of the disease to the spine is possible. Patients who are on antifungal therapy without a history of immunosuppression still have a risk of acquiring coccidioidomycosis. Identifying patients who have had a lapse of medical therapy through a thorough history taking will allow prompt medical and surgical management and may prevent further complications.
